# A CNN-LSTM Car-Following Model Considering Generalization Ability

**DOI:** 10.3390/s23020660

**Published:** 2023-01-06

**Authors:** Pinpin Qin, Hao Li, Ziming Li, Weilai Guan, Yuxin He

**Affiliations:** School of Mechanical Engineering, Guangxi University, Nanning 530004, China

**Keywords:** car-following, convolution neural network-long short-term memory, traffic flow theory, intelligent driving, generalization ability

## Abstract

To explore the potential relationship between the leading vehicle and the following vehicle during car-following, we proposed a novel car-following model combining a convolutional neural network (CNN) with a long short-term memory (LSTM) network. Firstly, 400 car-following periods were extracted from the natural driving database and the OpenACC car-following experiment database. Then, we developed a CNN-LSTM car-following model, and the CNN is employed to analyze the potential relationship between the vehicle’s dynamic parameters and to extract the features of car-following behavior to generate the feature vector. The LSTM network is adopted to save the feature vector and predict the speed of the following vehicle. Finally, the CNN-LSTM model is trained and tested with the extracted car-following trajectories data and compared with the classical car-following models (LSTM model, intelligent driver model). The results show that the accuracy and the ability to learn the heterogeneity of the proposed model are better than the other two. Furthermore, the CNN-LSTM model can accurately reproduce the hysteresis phenomenon of congested traffic flow and apply to heterogeneous traffic flow mixed with adaptive cruise control vehicles on the freeway, which indicates that it has strong generalization ability.

## 1. Introduction

Car-following is a crucial technology of intelligent driving systems. The car-following model with high accuracy and strong generalization ability is of great significance for driving safety, alleviating the psychological pressure of drivers in urban expressway congestion traffic flow, and lightening the operating burden of drivers on expressways.

Existing car-following models mainly include mathematical car-following models and data-driven car-following models according to the modeling method [1]. The mathematical car-following model is a quantitative analysis of car-following behavior based on the observation of it and careful consideration of vehicle dynamics, road conditions, and other factors to deduce an equation with clear physical parameters [2]. It has strong interpretability and two major problems.

(1) For different road types and traffic flows, they can only be applied after calibration, and their accuracy and generalization ability are limited [3]; and (2) with the increase in factors considered, the complexity of the model increases dramatically [4].

The data-driven car-following model is based on the trajectory data of the car-following. It takes advantage of data science and machine learning and investigates its internal rules through the study and summary of the data. Taking time as the boundary, data-driven car-following models mainly include traditional machine-learning, deep learning, and deep reinforcement learning car-following models.

The traditional machine-learning car-following model benefits from the rapid development of machine learning algorithms and the reduction of the difficulty of obtaining high-fidelity data, which establishes a foundation for developing the data-driven car-following model [5].

The deep learning car-following model mainly adopts recurrent neural network (RNN) and its improved algorithm. Zhou et al. [6] introduced the first RNN car-following model and compared it with the intelligent driver model (IDM), the results show that the RNN model can better reproduce the traffic oscillation and distinguish driver styles in traffic oscillation. Wang et al. [7] used the gated recurrent unit (GRU) of the RNN to develop a car-following model to predict the speed of the following vehicle at the next time step and compared it with the backpropagation neural network (BPNN) and IDM models through simulation. The results indicate that the GRU model has higher prediction accuracy. Wang et al. [8] established a deep neural network car-following model, the model performance with historical input information at different time scales was compared. The results imply that the car-following model should properly embed the long memory effect. Huang et al. [9] proposed an LSTM car-following model to study asymmetric driving behavior. The NGSIM database was used to conduct trajectory simulation and comparison between the RNN, full velocity difference, and LSTM models. The results show that the LSTM model had a better ability to capture asymmetric driving behavior. Ma et al. [10] proposed a sequence-to-sequence learning-based car-following model, which not only considered the memory effect but also the response delay. Through comparison experiments with the IDM and LSTM models, it was proved that this model could better reproduce car-following trajectories and heterogeneous driving behaviors.

The deep deterministic policy gradient (DDPG) algorithm is widely applied in the deep reinforcement learning car-following strategy because it is adept at addressing decision problems in continuous action space [11]. Zhu et al. [12] proposed a human-like autonomous car-following framework based on the DDPG algorithm. They used experimental data to conduct comparative experiments on the RNN, IDM, and DDPG models. The results show that the DDPG model has high prediction accuracy and good generalization ability. Then, Zhu et al. [13] combined a collision avoidance strategy with the DDPG algorithm to develop a speed control model for the following vehicle and used the model predictive control-based adaptive cruise control (MPC-ACC) model for comparative verification. The results show that the vehicles controlled by the DDPG model are superior to human drivers and the MPC-ACC model in terms of safety, comfort, and efficiency. Li et al. [14] proposed a deep reinforcement learning-based autonomous car-following decision-making strategy to improve the satisfaction and acceptance of automatic driving, and carried out extensive simulation experiments to validate the model’s effectiveness and accuracy.

The data-driven car-following model has promoted car-following development, and many achievements have been made. However, the following problems still exist.

(1) About 80% of the models adopt the NGSIM database, whose representativeness needs further verification [15]. (2) Existing data-driven car-following models are all trained and tested through a single database or driving scenario, and there is little analysis of their stability and generalization ability. (3) The car-following scenario in which human-driven and ACC vehicles are mixed on the freeway lacks attention. (4) The car-following model based on the DDPG algorithm excessively depends on the design of reward function, whose performance is unstable for different driving styles and traffic flows [11].

Therefore, we propose a novel car-following model combining a convolutional neural network (CNN) with the LSTM network that adapts between different car-following scenarios, road types, and traffic flows to provide more accurate vehicle dynamics than any prior model that we are aware of. The primary contributions of this study are: (1) Developing a new car-following model for congested traffic flow and mixed traffic flow; (2) providing a calibrated model which can achieve car-following simulation in multiple traffic flow types and share only one group of parameters; and (3) proving the accuracy and generalization ability of the proposed model. The rest of this paper is organized as follows. Section 2 introduces the data and the car-following trajectories data extraction standards. Section 3 develops the CNN-LSTM car-following model, which includes the combination of a CNN and the LSTM and model confifiguration. Section 4 carries out experiments and discussions, including calibration of models, model accuracy comparison, and generalization ability analysis. Section 5 concludes our findings.

## 2. Data Preparation

**Figure 1:**
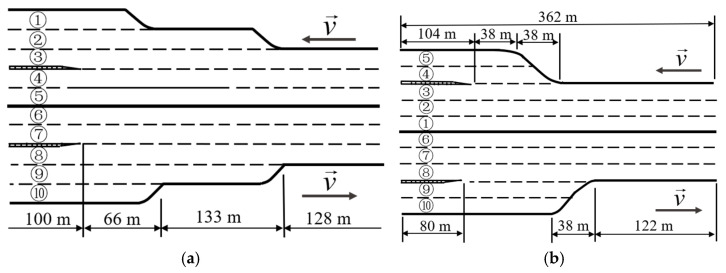
Schematic diagram of lane distribution, where (**a**) is dataset 1 and (**b**) is dataset 3.

The natural driving database of urban expressways was extracted by the UTE team of Southeast University using UAVs to shoot videos over four urban expressways [16]. Because the vehicles in dataset 2 are in a free-flow state, the duration of the car-following state is short; dataset 4 lacks vehicle number information, which cannot match the corresponding state parameters of vehicles. In this paper, datasets 1 and 3 are selected as the data sources of urban expressways. See Table 1 for the parameters of the database and Figure 1 for lane distribution.

OpenACC is an open database of car-following experiments to study the properties of commercial ACC systems [17], and the data of the first two experiments are used in this paper. Its parameters are shown in Table 2. For more information about the database, please refer to the research literature [17].

According to the following standards [7], 400 car-following periods were extracted from the two databases, and the cumulative duration is 3.682 × 10^4^ s.

(1) The leading vehicle and the following vehicle are in the same lane and the longitudinal gap is less than 100 m to prevent the following vehicles from running freely.

(2) To ensure that the vehicles do not change lanes or make sharp turns, the lateral distances’ difference between the leading vehicle and the following vehicle is less than 1.5 m.

(3) Each car-following period lasts for more than 30 s to ensure that the vehicle is in a stable car-following state.

## 3. Methodology

### 3.1. 1D CNN

**Figure 2:**
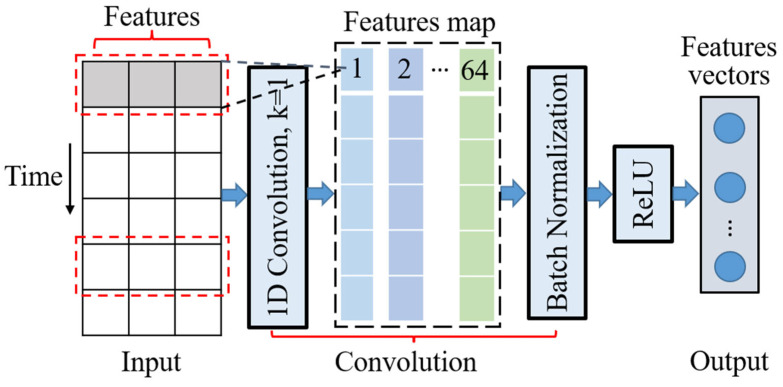
Feature extraction framework of 1D CNN.

Because of its outstanding feature extraction ability, 1D CNN is widely applied in time series data analysis [18], as shown in Figure 2, which includes an input layer, a convolution layer, and an output layer. The time series data are transferred into the convolution layer by the input layer, and the convolution kernel is adopted to extract the data features of the car-following trajectories, to capture the time dependence of the car-following behavior. At the same time, we adopt 64 convolutional kernels to integrate different features. The batch standardization method can accelerate the convergence speed and improve network performance. Furthermore, for the nonlinear attribute of car-following behavior, the activation function ReLU is introduced to fit the nonlinear relationship between the feature parameters to generate the feature vector.

### 3.2. LSTM Network

**Figure 3:**
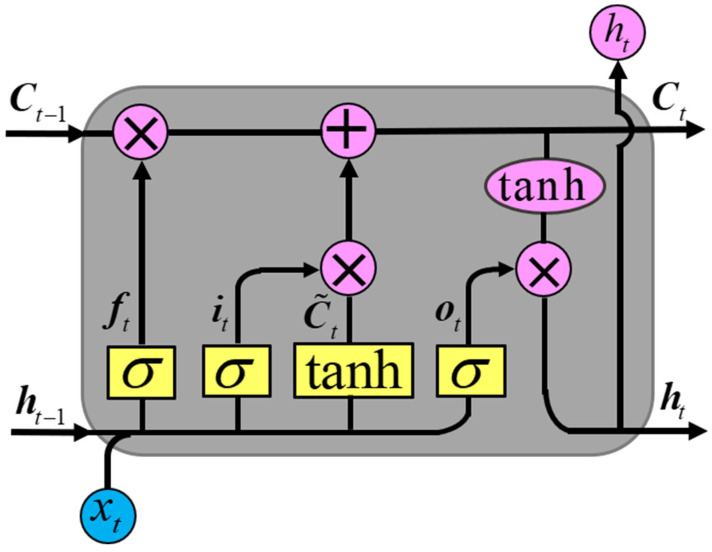
LSTM unit.

The LSTM unit is designed to settle the problem of gradient disappearance and gradient explosion in RNN during long-time series data processing [19]. As shown in Figure 3, it includes a forgetting gate ($f_{t}$), an input gate ($i_{t}$), and an output gate ($o_{t}$).

The forgetting gate can forget the information with a low correlation between the input quantity and the output quantity at the last moment to facilitate the subsequent remembering of new information. The forgetting ratio of the state information is calculated by the formula (1).

(1) $$f_{t}=\sigma \left(W_{f}\cdot \left[h_{t-1},x_{t}\right]+b_{f}\right)$$

where σ is the activation function, $W_{f}$ denotes the weight, $h_{t-1}$ is the output state at the last moment, $x_{t}$ denotes the input of the current time, and $b_{f}$ is bias.

The input gate selectively absorbs the useful information extracted from the forgetting gate and the information received at the current moment. The ratio of the current moment information to the selected memory and the information memorized by the memory cells are calculated by formula (2).

(2) $$\left.\begin{array}{l} i_{t}=\sigma \left(W_{i}\cdot \left[h_{t-1},x_{t}\right]+b_{i}\right)\\ {\tilde{C}}_{t}=\tanh\left(W_{C}\cdot \left[h_{t-1},x_{t}\right]+b_{C}\right)\\ C_{t}=f_{t}*C_{t-1}+i_{t}*{\tilde{C}}_{t} \end{array}\right\}$$

where tanh is the activation function, and $f_{t}*C_{t-1}$ and $i_{t}*{\tilde{C}}_{t}$ denote the retained past information and the remembered current information, respectively.

The output gate selects the updated information from the cells in the input gate and outputs it. The calculation formulae are:

(3) $$\left.\begin{array}{l} o_{t}=\sigma \left(W_{o}\cdot \left[h_{t-1},x_{t}\right]+b_{o}\right)\\ h_{t}=o_{t}*\tanh\left(C_{t}\right) \end{array}\right\}$$

where $o_{t}$ is the selection ratio of output information.

### 3.3. CNN-LSTM Car-Following Model

**Figure 4:**
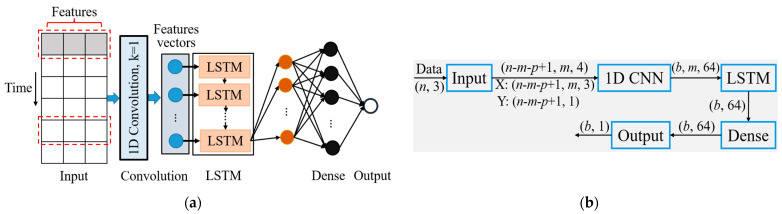
CNN-LSTM neural network car-following model structure, where (**a**) is the structure diagram of the proposed model, (**b**) is the flow of tensors in each layer, respectively. (*n*, *m*, *p*, and *b* are the number of samples, memory time step, prediction time step, and batch size, respectively).

The model mainly includes the 1D CNN module for extracting car-following behavior’s feature information, the LSTM network module for predicting the following vehicle’s speed, and an output module. The structure diagram of the CNN-LSTM neural network car-following model and its input and output dimensions of each layer are shown in Figure 4. Based on the data from the output module, the vehicle state is updated by formula (4).

(4) $$\left.\begin{array}{l} a_{f}\left(t\right)=\left[v_{f}\left(t+∆T\right)-v_{f}\left(t\right)\right]/∆T\\ x_{f}\left(t+∆T\right)=x_{f}\left(t\right)+v_{f}\left(t\right)\cdot ∆T+\frac{1}{2}a_{f}\left(t\right)\cdot ∆T^{2}\\ ∆x\left(t\right)=x_{l}\left(t\right)-x_{f}\left(t\right) \end{array}\right\}$$

where $a_{f}$ is the acceleration of the following vehicle at time *t*, ∆T denotes the sampling time step (0.08 s and 0.1 s, respectively), $v_{f}$ is the speed of the following vehicle at time *t*, and $x_{l}\left(t\right)$ and $x_{f}\left(t\right)$ are the longitudinal positions of the leading vehicle and the following vehicle at time *t*.

### 3.4. Configuration of CNN-LSTM Model

Car-following behavior varies with different road types and traffic conditions. Configuring the model according to observed vehicle trajectory data and road conditions is conducive to improving the model’s ability to reproduce car-following behavior.

#### 3.4.1. Input and Output Variables

During car-following, the following vehicle’s driver adjusts the vehicle’s speed according to the driving state of the leading vehicle. To explore the factors that affect the speed of the following vehicle, we carried out Pearson and Spearman correlation analyses to conduct a correlation study on each parameter. The results showed that for the correlation coefficient between the following vehicle’s speed and the gap between the leading vehicle and the following vehicle (∆x), the relative velocity (∆v) was significant. Therefore, $v_{f}\left(t\right),\;∆x\left(t\right)$, and $∆v\left(t\right)$ were selected as the input variables, $v_{f}\left(t+\left(p-m\right)\cdot ∆T\right)$ as the output variables (*m*, *p* represent the memory time step and prediction time step).

To reduce the influence of the difference of an order of magnitude between different variables on the model’s learning speed and training effect, formula (5) is used to normalize each variable.

(5) $$u^{\prime }=\frac{u-u_{\min}}{u_{\max}-u_{\min}}$$

where $u^{\prime }$ denotes the normalized variable, whose value range is [0, 1], *u* is the variable before normalization, $u_{\max},\;u_{\min}$ are the maximum and minimum values of variables, respectively.

#### 3.4.2. Optimization Algorithm and Activation Function

An Adam algorithm was selected as the optimization algorithm of the CNN-LSTM car-following model. The optimization algorithm can effectively update the network weight and convergence speed. The activation function can retain the features learned by neurons in the form of a function and map them to the model’s output. ReLU is chosen as the activation function of the model [9].

#### 3.4.3. Loss Function

The loss function is used to evaluate the consistency between the simulated value generated by the model and the observed value. The more reasonable the design of the loss function, the better the model’s performance. We adopt the mean square error (MSE) of the following vehicle’s speed as the loss function.

(6) $$F_{\mathrm{loss}}=MSE(v_{f})=\frac{1}{n}\sum_{i=1}^{n}{\left[v_{f}^{\mathrm{sim}}\left(i\right)-v_{f}^{\mathrm{obs}}\left(i\right)\right]}^{2}$$

where *n* is the number of samples, and $v_{f}^{\mathrm{obs}}\left(i\right),\;v_{f}^{\mathrm{sim}}\left(i\right)$ represent the *i*-th observed value and predicted value of the following vehicle’s speed, respectively.

#### 3.4.4. Metrics of the Model Performance

The metric is used to evaluate the model’s prediction performance, and the mean absolute percentage error (MAPE) of the following vehicle’s speed is adopted as the performance measurement index of the model.

(7) $$MAPE_{v_{f}}=\frac{1}{n}\sum_{i=1}^{n}\left|\frac{v_{f}^{\mathrm{sim}}\left(i\right)-v_{f}^{\mathrm{obs}}\left(i\right)}{v_{f}^{\mathrm{obs}}\left(i\right)}\right|$$

#### 3.4.5. Memory Time Step

The literature research [8] shows that the driver’s memory effect should be considered in the car-following model. The memory time step of the proposed method is preset as {5,10,15}, and the model’s performance in the training process determines the final memory time step.

#### 3.4.6. Prediction Time Step

The existing car-following models based on deep learning algorithms predict the output variable of the next time step, and the driving decisions in the observed car-following process are completed in multiple time steps with response delay [10]. To determine the prediction time step of the model, we employed the cross-validation method to find the best prediction time step in the model calibration process.

## 4. Experimental Results and Discussion

**Figure 5:**
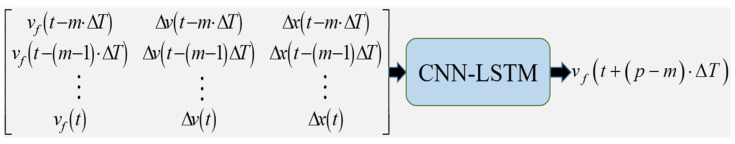
Framework overview.

The drive to develop an excellent car-following model arises from the need to analyze the traffic flow effects of proposed road network changes. Most car-following models in current use can be described as formula (8) [20]. The framework overview of the proposed model is shown in Figure 5. It obtains a better mapping relationship among the dynamic parameters by learning and summarizing the car-following trajectory data. The vehicle state changes with its acceleration, and the vehicle location can be updated according to formula (4).

(8) $$a_{f}\left(t+\tau \right)=l_{n}\frac{{\left[v_{l}\left(t\right)-v_{f}\left(t\right)\right]}^{k}}{{\left[x_{l}\left(t\right)-x_{f}\left(t\right)\right]}^{m}}$$

where τ is the reaction time, and $l_{n},\;k$, and m are parameters that need to be estimated.

To verify the performance of the proposed model, we compare it with the IDM and LSTM models based on the same experiment. To our knowledge, the LSTM model is the best deep learning car-following model nowadays. IDM is a widely used mathematical car-following model [8]. It assumes that acceleration is a continuous function of the velocity, gap, and velocity difference. IDM can be described as formulae (9).

(9) $$\left.\begin{array}{l} a_{\mathrm{IDM}}=a\left[1-{\left(\frac{v_{f}\left(t\right)}{v_{0}}\right)}^{\delta }-{\left(\frac{s^{*}\left(v_{f}\left(t\right),∆v\left(t\right)\right)}{∆x}\right)}^{2}\right]\\ s^{*}\left(v_{f}\left(t\right),∆v\left(t\right)\right)=s_{0}+v_{f}\left(t\right)\cdot T+\frac{v_{f}\cdot ∆v\left(t\right)}{2\sqrt{ab}} \end{array}\right\}$$

where $a_{\mathrm{IDM}}$ is the acceleration calculated by IDM, $v_{0}$ denotes the expected speed of the following vehicle, $s^{*}\left(v_{f}\left(t\right),∆v\left(t\right)\right)$ is the safe gap calculated by IDM at time *t*, $s_{0}$ denotes the safe distance of the vehicle at rest (its default value is 2 m), δ is the acceleration index (its default value is 4), T denotes the time headway, and a, b are the maximum acceleration and comfortable deceleration.

### 4.1. Calibration of Models

We randomly selected 70% (280 periods) of the extracted data to train the CNN-LSTM car-following model, and the remaining data were used as the test dataset. The cross-validation method is employed to calibrate the memory time step, prediction time step, number of LSTM layers, number of full connection layers, number of neurons, batch size, and epochs. The calibration results are shown in Table 3. The LSTM and CNN-LSTM models adopt the same configuration and parameters, as shown in Table 4. Different road conditions, traffic flows, and drivers’ driving styles lead to different parameter calibration results of the IDM. Therefore, they are all an interval rather than a constant, as shown in Table 5.

### 4.2. Accuracy

The accuracy of the model means that the model can efficiently study and summarize the driver’s driving habits and predict future driving behavior by learning the car-following trajectory data. The car-following model with high accuracy can reproduce complex traffic phenomena and promote the research of traffic simulation.

**Figure 6:**
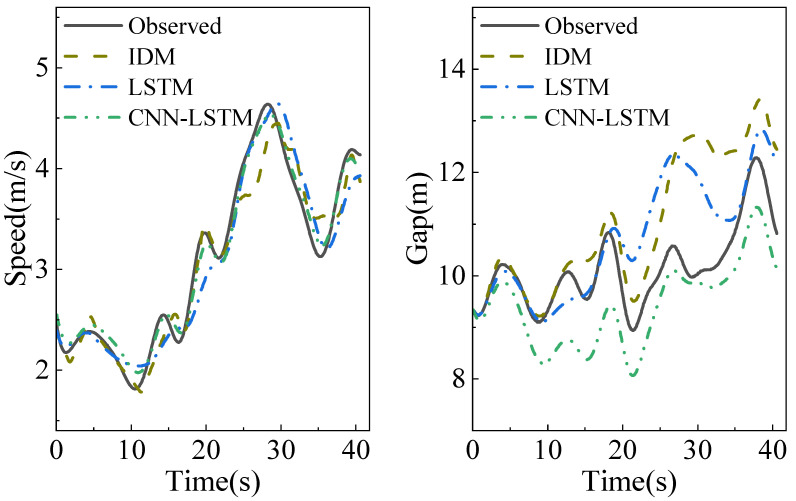
Performance comparison of 3 models.

The statistical results of 100 test periods’ errors are shown in Table 6. The speed is low and the acceleration variation is considerable in the natural driving database, which can reflect the drivers’ features during car-following. The speed is high, the acceleration variation is slight, and the data quality is high in the OpenACC database. Therefore, the simulation errors of the three models in the OpenACC database are significant. The error distribution range and the mean of the IDM are more significant than the other two, which indicates that the neural network models have strong stability. Although there are differences in road conditions and traffic flow types, compared with the IDM and LSTM models, the CNN-LSTM model reduces the mean MSE of speed simulation by 76.0% and 55.3%, respectively, which shows that it has high accuracy and strong migration ability. We randomly selected one period from the test results to further analyze the differences between the three models. Figure 6 shows the simulation results of the three models corresponding to this period. Compared with the LSTM model, the speed simulation value generated by the CNN-LSTM model retains a minor error with the observed value and accurately captures the changing trend of the speed at all times, highlighting the importance of feature extraction. 

### 4.3. Generalization Ability

A neural network’s ability to use what it has learned from previous experiences to operate in a completely new environment is called generalization ability [21]. The car-following model with strong generalization ability can be applied to different road conditions, vehicle types, and traffic flows. In this paper, the generalization ability of each model is evaluated by its ability to learn heterogeneity, reproduce the hysteresis phenomenon of congested traffic flow, and adapt the heterogeneous traffic flow mixed with ACC vehicles.

**Figure 7:**
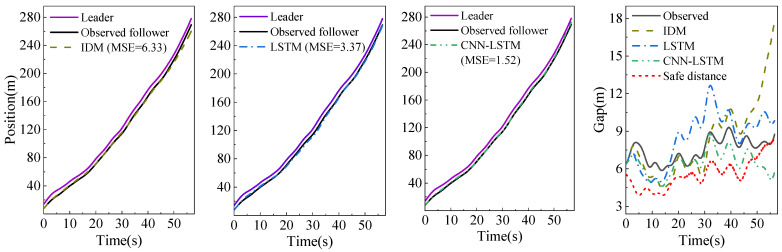
Aggressive driving behavior.

**Figure 8:**
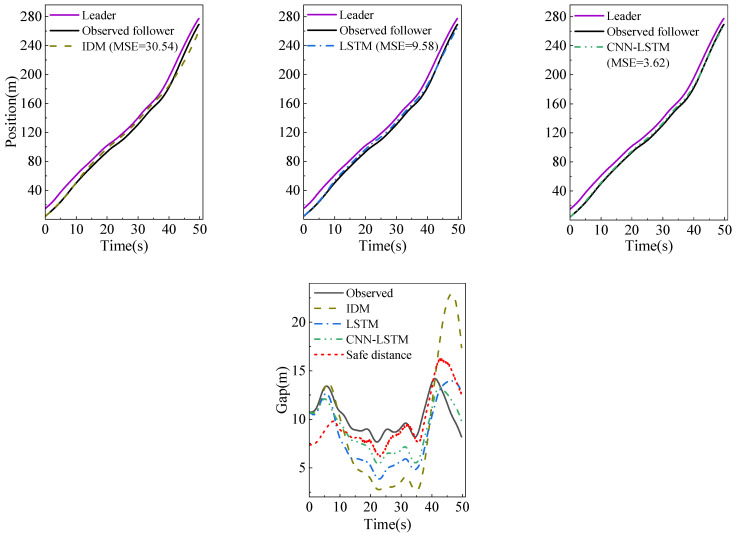
Normal driving behavior.

Heterogeneity includes the heterogeneity of driving behavior (different driving styles) and the heterogeneity of drivers (human, ACC system). We adopted the *K*-means algorithm to cluster the mean gap and the standard deviation of the following vehicles’ speeds. The driving styles are divided into aggressive and normal [12]. We randomly selected the periods of two driving types from the test dataset. The experimental results are shown in Figure 7 and Figure 8. Due to error accumulation, the IDM generates significant gap errors in the later stage of the car-following process. The deep learning car-following model is good at learning and summarizing the rules of the trajectories data, resulting in a small error between the generated trajectories and the observed trajectories, indicating that the deep learning algorithm has a strong ability to learn heterogeneous driving behaviors. Compared with the LSTM model with the same configuration and parameters, the CNN-LSTM model can reduce the trajectory prediction error by 54.9% and 62.2%, respectively. Moreover, it can reduce the running time by 60.8% compared with the LSTM model, which verifies the effectiveness of the CNN in the feature extraction of car-following behavior. It is known from the generalized force model that the driver of the following vehicle expects to keep a safe distance positively related to the speed of his vehicle with the leading vehicle during car-following [22]. As shown in formula (10), we employ it as the safety distance, and the time headway corresponding to different driving styles. Each model’s simulation gap and the safe distance are shown in the fourth subgraphs of Figure 7 and Figure 8. All models cannot guarantee that the simulation gap is higher than the safe distance, but the simulation gap generated by the CNN-LSTM model is the closest to the safe distance, which indicates that the proposed model can better reproduce the driver’s expected following behavior.

(10) $$s\left(v_{f}\right)=v_{f}\left(t\right)\cdot T+d$$

where s is the safe distance, T is the time headway (the time headways of aggressive and ordinary drivers are 0.8 s and 1.5 s, respectively), and d is the minimum vehicle distance (its default value is 2 m).

In modeling car-following behavior, reproducing some unexplained traffic phenomena is the key to measuring the model’s performance. This experiment evaluates the ability of the model to produce the hysteresis phenomenon by the platoon simulation of five vehicles in a congested traffic flow with three models. The trajectory of the first following vehicle is determined by the observed course of the leading vehicle and its initial condition. Additionally, the trajectory of the next following vehicle is generated by the simulation trajectory of the leading vehicle and its initial state. However, the simulated trajectory of the last following vehicle has a significant error with the observed trajectory because of the cumulative error. 

**Figure 9:**
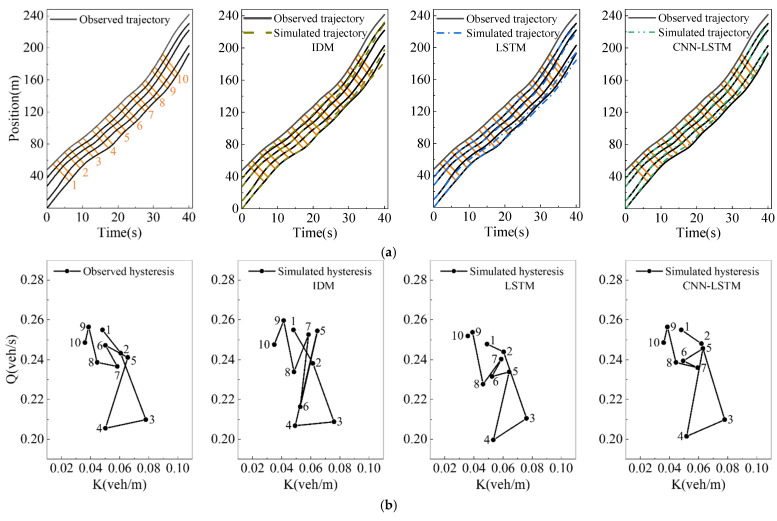
Platoon simulation in congested traffic flow, where (**a**) is the trajectory comparison, (**b**) is the flow-density diagram, respectively.

Figure 9b shows the traffic oscillation scenario in which the clockwise and counterclockwise hysteresis phenomena co-occur in congested traffic flow. We use Laval’s aggregation method [23] to analyze it, which calculates the traffic flow and density through the parallelogram in the time-space graph, and draws a polygonal line graph to show the hysteresis loop. In Figure 9a, the orange parallelogram has two long sides, the slope represents the wave propagation speed, and the slope of the two short sides is the vehicle speed. The traffic flow and density in each parallelogram area are calculated by formula (11).

(11) $$\left.\begin{array}{l} k=\sum_{i=1}^{n}t_{i}/\left|A\right|\\ q=\sum_{i=1}^{n}x_{i}/\left|A\right| \end{array}\right\}$$

where k and q represent the density and flow in parallelogram area A, respectively, $t_{i},\;x_{i}$ are the travel time and distance of the *i*-th vehicle passing through area A, respectively, n represents the number of vehicles in area A(n≥5), and $\left|A\right|$ is the area of A.

As shown in Figure 9a, the simulation trajectory of the last vehicle in the platoon may be outside the parallelogram. To ensure the parallelogram area remains unchanged and to calculate the traffic flow and density close to the observed traffic, we use the observed trajectory of the last vehicle instead of its simulation trajectory to calculate the flow and density [24].

Figure 9b shows the hysteresis loops corresponding to the simulation trajectories of the three models. We can find that the three models can accurately reproduce the clockwise hysteresis loops corresponding to areas 1 to 5. However, the counterclockwise hysteresis loops corresponding to areas 5 to 7 are only shown in the trajectory simulated by the CNN-LSTM model, which shows that the proposed model can not only reduce the cumulative error of the platoon simulation, but accurately extract the features of the platoon to reproduce the traffic hysteresis phenomenon.

**Figure 10:**
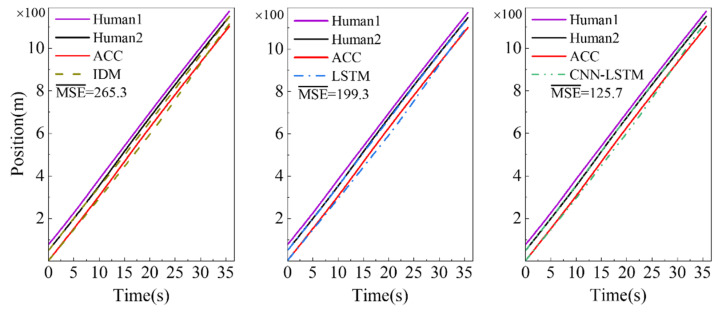
Platoon simulation in heterogeneous traffic flow mixed with ACC vehicles.

To further test the performance of adapting to the heterogeneous traffic flow mixed with ACC vehicles, we carry out a car-following test in a high-speed scenario containing two human-driven vehicles and one vehicle controlled by an ACC system. The results are shown in Figure 10. There are apparent errors between the simulation trajectories generated by the IDM and the observed trajectories. The vehicle controlled by the ACC system accelerates from 17 to 30 s, and the simulation trajectories of the three models are hysteretic. Still, the simulation error of the CNN-LSTM model is the smallest, which reflects that it has a strong adaptability.

## 5. Conclusions

This paper develops a car-following model based on a CNN and the LSTM network to improve its accuracy and generalization ability. The proposed model was trained and tested through the natural driving database and OpenACC databases, and it was compared with the IDM and LSTM models. Compared with the LSTM model with the same configuration parameters, the CNN-LSTM model can decrease the mean square error of speed simulation of the single car-following pair by 55.3% and reduce the mean simulation trajectories error of the platoon by 36.9% in heterogeneous traffic flow mixed with adaptive cruise control vehicles. Furthermore, the CNN-LSTM model can reduce the mean simulation trajectories error of the platoon by 60.9% in congested traffic flow. The results proved the effectiveness of the CNN for car-following behavior feature extraction. Moreover, the CNN-LSTM model can accurately reproduce heterogeneous driving behavior and the hysteresis phenomenon of congested traffic flow, highlighting its strong learning ability and accuracy. For different traffic flows and road conditions, the CNN-LSTM model can perform better than the other two, proving that it has strong generalization ability.

In addition, the simulation gap generated by the CNN-LSTM model is the closest to the safe distance, which indicates that the proposed model can better reproduce the driver’s expected following behavior. However, how to combine the safety issue with the deep learning car-following model is still an urgent problem to be addressed. Furthermore, the simulation speed curve generated by the proposed model is smooth, which can improve vehicular ride comfort. Road transportation efficiency and fuel economy can be improved by integrating ease traffic congestion into the car-following model. This requires more effort and experimental research.

## Figures and Tables

**Table 1:** Introduction of natural driving vehicle trajectory database.

Parameter	Value	
	Dataset 1	Dataset 3
Road length (m)	427	362
Duration (s)	255	545
Temporal accuracy (s)	0.01	
Position accuracy (m)	0.01	
Sampling frequency (Hz)	25	

**Table 2:** Introduction of the OpenACC database.

Parameter	Value			
Road type	Freeway			
Campaign	1		2	
Driving mode	Human	ACC	Human	ACC
Duration (h)	5.70	5.28	4.31	5.69
Distance covered (km)	569	519	399	602
Sampling frequency (Hz)	10			

**Table 3:** Parameters of the CNN-LSTM car-following model.

Parameter	Value
Number of convolutional kernels	64
Number of convolution layers	1
Batch size	128
Memory time step	5
Prediction time step	10
Number of LSTM layers	1
Number of fully connected layers	1
Number of neurons in the LSTM layer	64
Epochs	60

**Table 4:** Parameters of the LSTM car-following model.

Parameter	Value
Batch size	128
Memory time step	5
Prediction time step	10
Number of LSTM layers	1
Number of fully connected layers	1
Number of neurons in the LSTM layer	64
Epochs	60

**Table 5:** Parameters of the IDM.

Parameter	Freeway	Urban Expressway
Desired speed (m/s)	[34, 38]	[10, 20]
Time headway (s)	[0.6, 3.8]	[0.7, 1.7]
Maximum acceleration (m/s^2^)	[0.9, 1.4]	[1.2, 2.1]
Comfortable deceleration (m/s^2^)	[0.8, 1.6]	[0.9, 2.5]

**Table 6:** Statistical results of speed simulation error.

Database	Model	MSE		
		Minimum	Mean	Maximum
Natural driving database	IDM	0.098	0.35	1.334
	LSTM	0.035	0.135	0.506
	CNN-LSTM	0.023	0.084	0.305
OpenACC database	IDM	0.143	2.683	27.546
	LSTM	0.041	1.480	20.855
	CNN-LSTM	0.009	0.662	4.740

## Data Availability

Not applicable.
